# Trans‐Tendon Suture‐Bridge Knotless Proximal Pulley Repair With Bioinductive Patch Augmentation for High‐Grade Partial Hip Abductor Tendon Tears

**DOI:** 10.1002/atn2.70142

**Published:** 2026-06-15

**Authors:** Maria Agustina Olarán, Napatpong Thamrongskulsiri, Felipe Casanova, Tomás F. Vega, Jorge Sánchez‐Mateos, Tanner Nishioka, Jorge Chahla

**Affiliations:** ^1^ Department of Orthopaedics Rush University Medical Center Chicago Illinois U.S.A.; ^2^ Servicio de Ortopedia y Traumatología Hospital Británico de Buenos Aires Buenos Aires Argentina; ^3^ Sports Medicine Research Group, Faculty of Medicine Chulalongkorn University Bangkok Thailand; ^4^ Servicio de Ortopedia y Traumatología Hospital Universitario La Paz Madrid Spain

## Abstract

This technical note describes a knotless trans‐tendon proximal‐pulley repair with distal suture‐bridge fixation and bioinductive collagen patch augmentation for high‐grade partial gluteus medius and minimus tears. The technique preserves intact tendon fibers, restores broad footprint compression, and supports biologic healing through a resorbable collagen scaffold. Detailed steps emphasize anatomic anchor placement, controlled tensioning, and efficient suture management to improve construct reliability. This combined mechanical and biologic augmentation offers a promising option for enhancing tendon healing and optimizing outcomes in patients with degenerative high‐grade partial‐thickness abductor tears.

## Graphical abstract

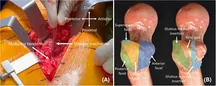

**VIDEO 1:** Video description of technique for completing a trans‐tendon suture bridge knotless proximal pulley repair with bioinductive collagen patch augmentation for high‐grade partial hip abductor tendon tears with 3 proximal all‐suture anchors (2.6‐mm FiberTak RC, Arthrex, Naples, FL) loaded with 1.3‐mm SutureTapes (Arthrex, Naples, FL) and 2 distal hard body anchors (4.75 mm PEEK SwiveLock, Arthrex, Naples, FL). Video content can be viewed at https://doi.org/10.1002/atn2.70142.

Hip abductor tendon tears of the gluteus medius and minimus are a major cause of lateral hip pain and dysfunction, most commonly degenerative and compromising abductor stability during gait and single‐leg stance.^1^ Improved imaging now detects both full‐ and partial‐thickness tears, including undersurface lesions, with prevalence ranging from 20% to 25% in arthroplasty patients to nearly 50% of those with refractory lateral hip pain.^2^, ^3^, ^4^

Despite advances in open and endoscopic techniques, outcomes after gluteus medius and minimus repair remain variable, with persistent weakness, Trendelenburg gait, and delayed recovery reported in a substantial proportion of patients.^2^, ^4^, ^5^ In some series, up to 20% of patients fail to reach acceptable symptom states or return to activity by 2 years, highlighting persistent challenges associated with tendon degeneration and limited biologic healing potential.^5^, ^6^

Rotator‐cuff literature for structurally analogous partial articular‐sided supraspinatus tendon avulsions supports trans‐tendon repair, which preserves intact fibers, maintains native length‐tension, and improves footprint compression.^7^, ^8^, ^9^, ^10^, ^11^ Combining these technical principles with bioinductive collagen patch augmentation provides a biomechanically and biologically advantageous option to address the limited healing potential of degenerative gluteal tissue.

This technical note describes a knotless trans‐tendon proximal‐pulley repair with distal‐row fixation and bioinductive patch augmentation to optimize healing and construct strength for partial‐thickness gluteus medius and minimus tears.

## SURGICAL TECHNIQUE

### Patient Positioning

After anesthesia is administered, the patient is positioned in the lateral decubitus position with the operative side up. The limb is then prepped and draped in sterile fashion, and a timeout is performed. A padded Mayo stand is placed to support the foot, allowing slight abduction to reduce tension on the iliotibial band throughout the procedure (Video 1).

### Surgical Approach

An 8‐cm longitudinal incision centered over the greater trochanter is made. Sharp dissection is carried down to the deep fascia and iliotibial band. The iliotibial band and deep fascia are then incised longitudinally in line with the long axis of the femur over the prominence of the greater trochanter to expose the trochanteric bursa, with care not to injure the underlying abductor tendons. The bursa is debrided, allowing visualization of the gluteus medius and minimus tendons. The tendons are grasped with forceps, confirming that the tissue is thinned and partially torn from the undersurface (Figure 1A).

**FIGURE 1:**
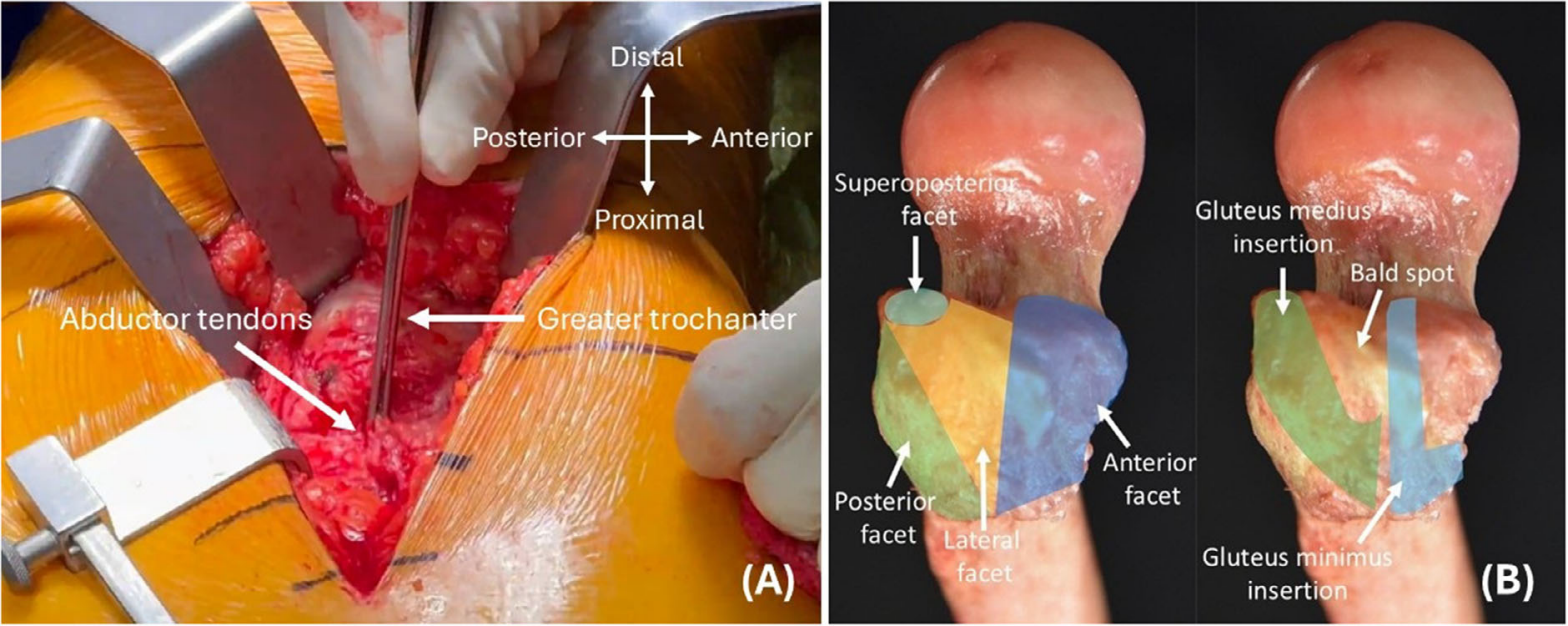
Exposure of the left hip in the lateral decubitus position. (A) Through an 8‐cm lateral incision, the iliotibial band and trochanteric bursa are opened longitudinally to visualize the gluteus medius and minimus tendons. Thinning and undersurface tearing of the abductor tendons are confirmed on direct inspection. (B) Anatomic footprint of the abductors on the greater trochanter is shown: the gluteus minimus attaches to the anterior facet, whereas the gluteus medius inserts onto the lateral and superoposterior facets, guiding the 3‐point anchor configuration.

### Placement of the Suture Anchors

The gluteus medius inserts onto the superoposterior and lateral facets of the greater trochanter, whereas the gluteus minimus attaches to the anterior facet (Figure 1B). During anchor placement, limb rotation can greatly assist with visualization; external rotation exposes the anterior facet more clearly, while internal rotation improves access to the posterior facets. After achieving optimal visualization, the first knotless FiberTak 2.6‐mm all‐suture anchor loaded with 1.3‐mm SutureTapes (Arthrex, Naples, FL) is placed at the anterior facet. The drill guide is positioned through the tendon to engage the bone, the pilot hole is created, and the anchor is inserted (Figure 2A). The second anchor is then placed at the lateral facet, followed by a third anchor at the superoposterior facet of the greater trochanter, completing a triangular anchor arrangement spanning the native gluteus medius and minimus footprint (Figure 2B,C).

**FIGURE 2:**
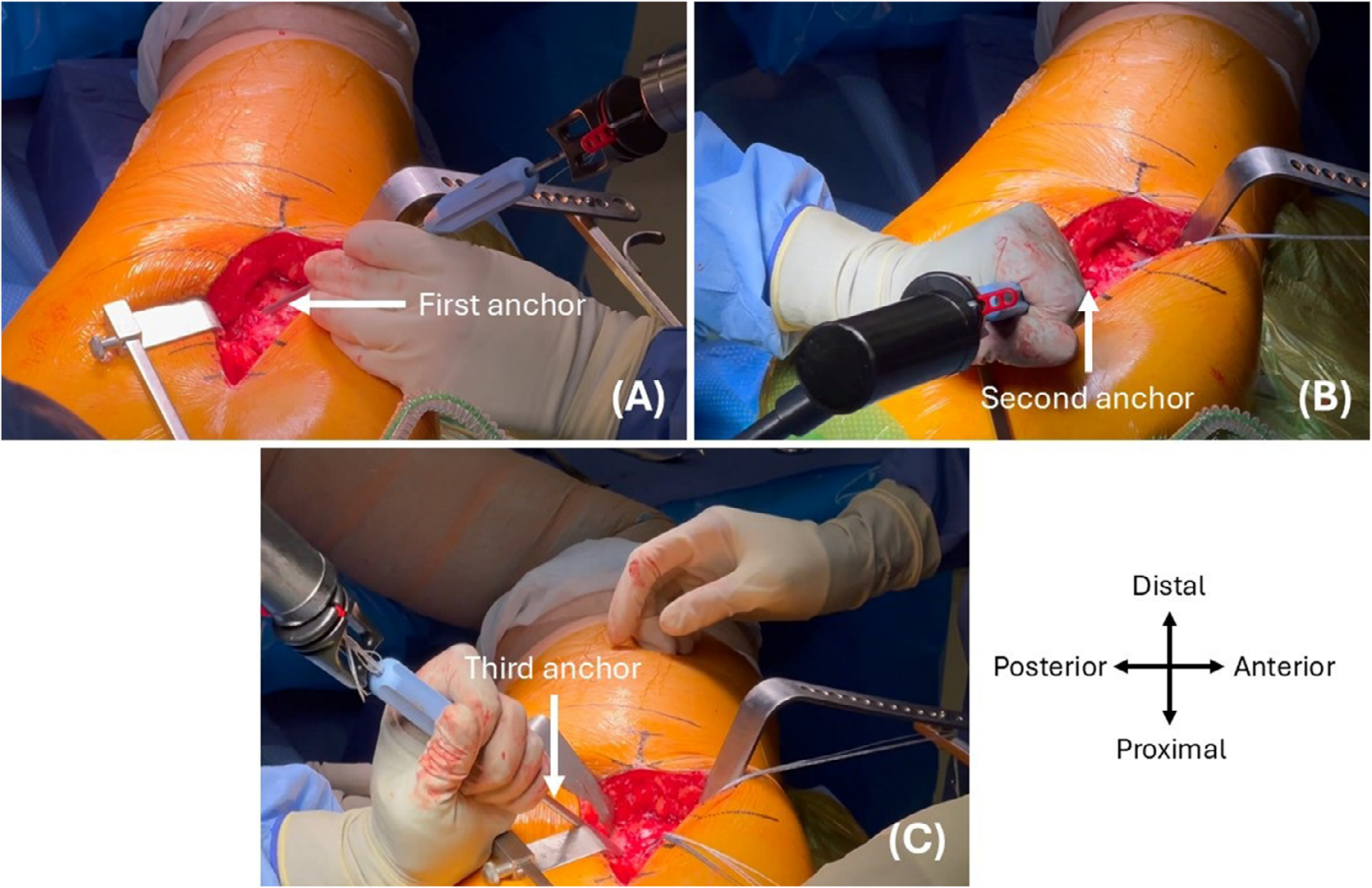
Anchor placement in the left hip with the patient in the lateral decubitus position. (A) The first FiberTak all‐suture anchor (Arthrex, Naples, FL) is inserted at the anterior facet using a drill guide passed through the tendon to engage bone, followed by pilot‐hole creation and anchor deployment. (B) The second anchor is positioned at the lateral facet. (C) The third anchor is placed at the superoposterior facet, completing a triangular anchor arrangement spanning the native gluteus medius and minimus footprint.

### Creating the Proximal Pulleys

The repair limb from the first anchor is passed through the loop from the second anchor. The conversion limb is shuttled through to activate the knotless loop mechanism, creating the first proximal pulley, which is left in place for later fixation of the bioinductive patch. Next, the repair limb from the second anchor is passed through the loop of the third anchor, and the conversion limb is shuttled to engage the knotless mechanism and create the second proximal pulley. Finally, the repair limb from the third anchor is passed through the loop of the first anchor, and the conversion limb is shuttled to activate the knotless mechanism and form the third pulley, but the repair limbs are not tensioned yet. Importantly, the 3 remaining sutures from the repair limbs of each proximal row anchor are not cut at this point, as these will be utilized for subsequent patch compression and tendon fixation along with the SutureTapes (Figure 3). The 3 anchors’ repair limbs and six 1.3‐mm SutureTape limbs (1 and 2 per proximal row anchor, respectively) emerging from each suture anchor will then be used to create the trans‐tendon suture bridges by connecting with the distal row SwiveLock anchors (Arthrex, Naples, FL).

**FIGURE 3:**
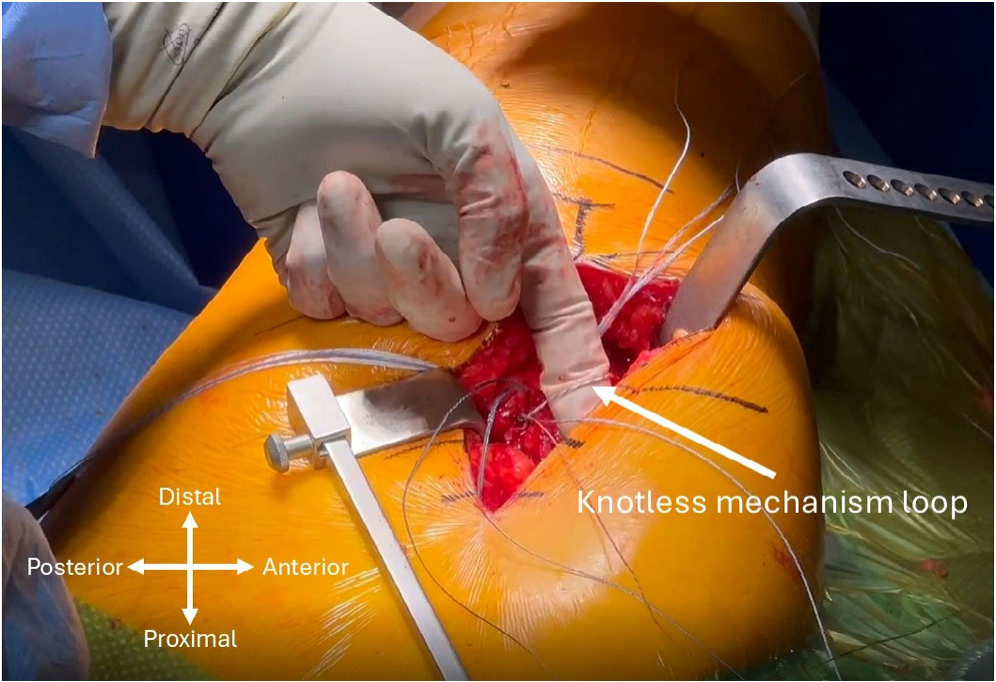
Creation of the knotless proximal pulley loops in the left hip with the patient in the lateral decubitus position. The repair limb of each anchor is passed through the loop limb of the next anchor. Shuttle tensioning of each loop limb activates the knotless mechanism, producing 3 independent proximal pulley loops for subsequent patch compression and tendon fixation.

### Placement of the Bioinductive Patch

The bioinductive patch (REGENETEN; Smith & Nephew, Andover, MA) is positioned over the abductor tendons and beneath the previously created loops (Figure 4A). Each loop is now tensioned to cinch down and compress the patch and the torn abductor tendons against the footprint, providing stable compression for biologic augmentation (Figure 4B).

**FIGURE 4:**
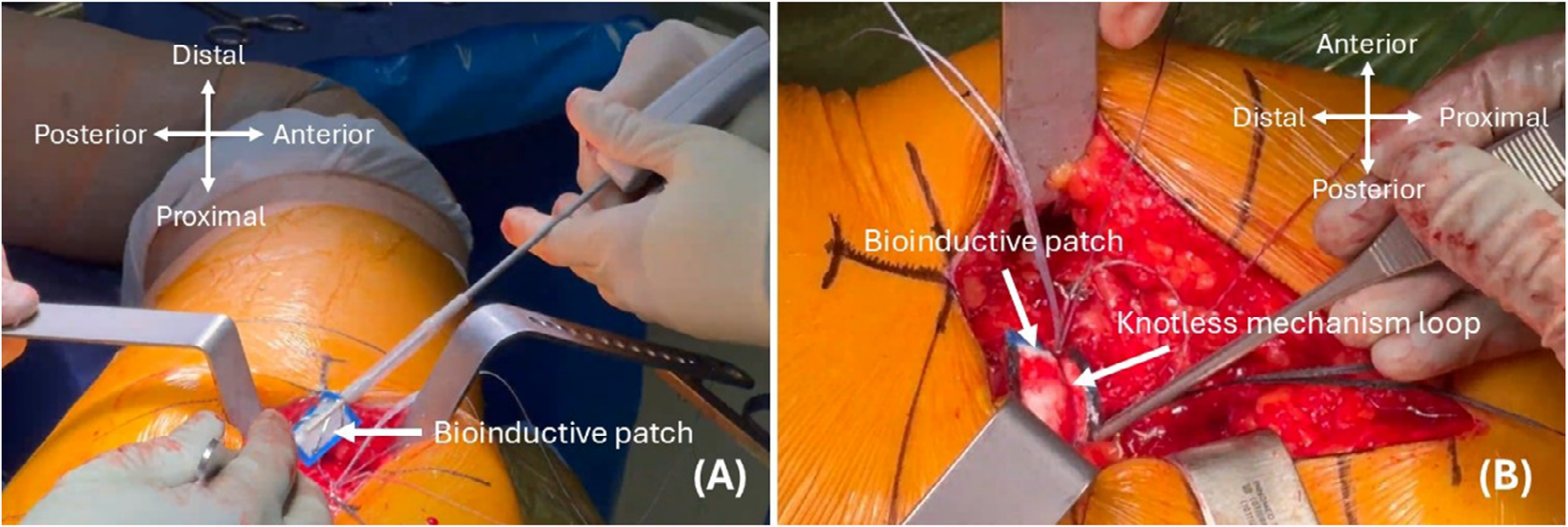
Placement and fixation of the bioinductive patch in the left hip in the lateral decubitus position. (A) The REGENETEN bioinductive patch (Smith & Nephew, Andover, MA) is positioned over the gluteus medius and minimus tendons and beneath the previously constructed pulley loops. (B) Tensioning of the pulley loops cinches the patch securely onto the tendon footprint, providing uniform compression and biologic augmentation.

### Distal‐Row Suture‐Bridge Fixation

The remaining suture from the 3 repair limbs and the 6 limbs of free SutureTape from the proximal row are then used to create a suture‐bridge construct with the anchors from the distal row. Two pilot holes are prepared on the lateral aspect of the greater trochanter, 1 anterior and 1 posterior, using a 4.75‐mm drill, and each hole is tapped to the same size (Figure 5A,B).

**FIGURE 5:**
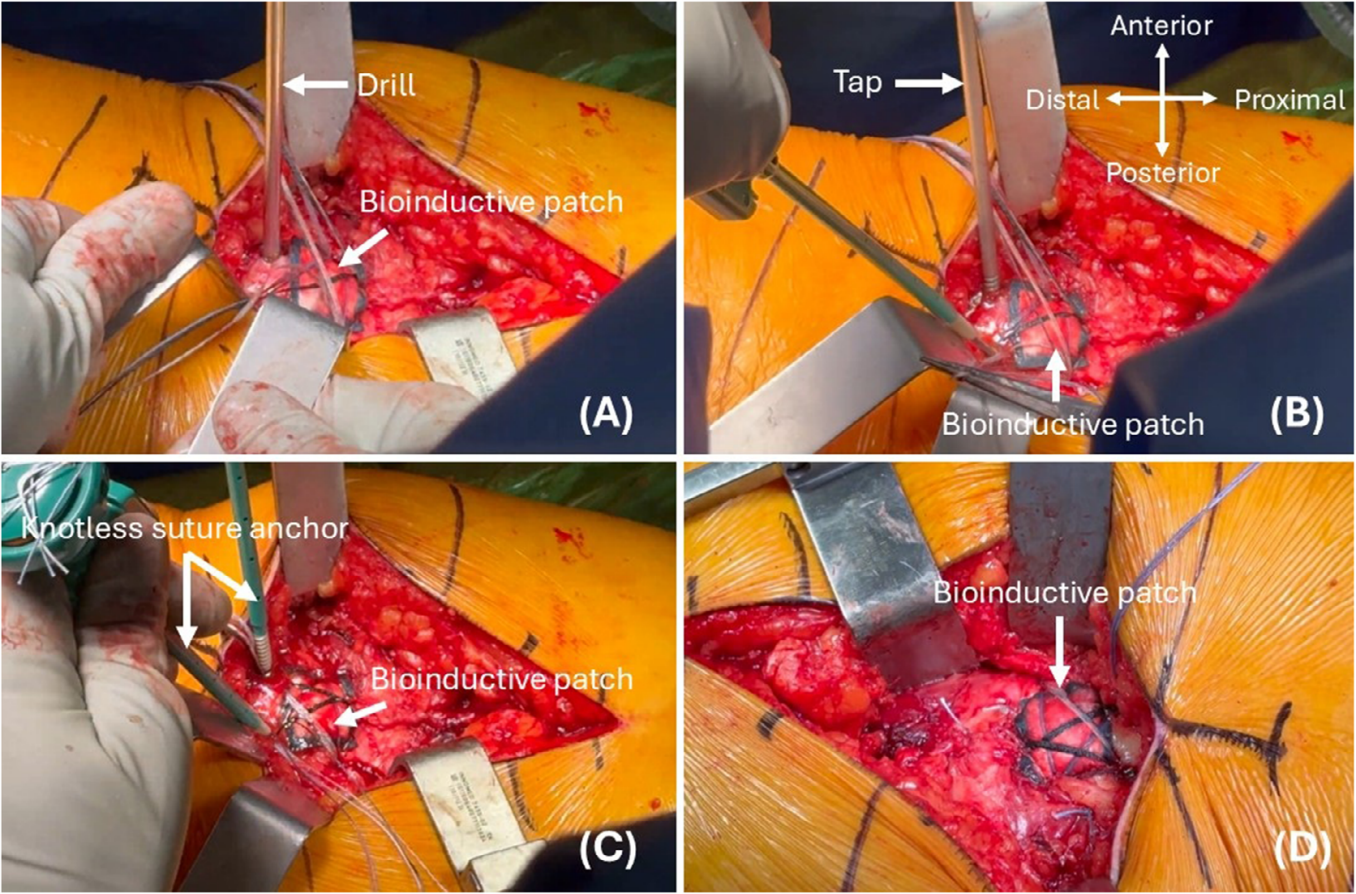
Distal‐row suture‐bridge fixation in the left hip with the patient in the distal decubitus position. (A,B) Two 4.75‐mm pilot holes (anterior and posterior) are drilled and tapped on the lateral aspect of the greater trochanter. (C) A 4.75‐mm SwiveLock knotless anchor (Arthrex, Naples, FL) is loaded with 1 limb from each FiberTak anchor (Arthrex, Naples, FL) and inserted into the anterior hole, followed by placement of a second SwiveLock (Arthrex, Naples, FL) in the posterior hole. (D) Final tensioning and seating create a robust anterior‐posterior suture‐bridge construct that compresses the bioinductive patch and repaired tendons against the footprint, completing the knotless trans‐tendon pulley‐bridge repair.

A 4.75‐mm SwiveLock knotless anchor (Arthrex, Naples, FL) is loaded with 1 repair limb from each of the 3 suture anchors (two in one and one in the other), and 1 limb of SutureTape from each knotless FiberTak (3 per SwiveLock) is loaded into the hard body anchors and inserted into the anterior pilot hole. The same process is repeated for the posterior anchor (Figure 5C). The trans‐tendon suture bridge construct is tensioned before final seating of each anchor to compress the bioinductive patch and tendon onto the footprint, completing a robust anterior‐posterior suture‐bridge construct that enhances compression of the patch and underlying tendon (Figure 5D).

## DISCUSSION

In this technical report, we describe a knotless trans‐tendon proximal‐pulley repair combined with distal‐row suture‐bridge fixation and bioinductive collagen patch augmentation for high‐grade partial gluteus medius and minimus tears, borrowing principles from partial articular‐sided supraspinatus tendon avulsions repairs of the rotator cuff. This knotless pulley‐bridge construct improves contact area while reducing the knot stack, and the bioinductive scaffold enhances the healing environment of degenerative tendons.^12^, ^13^, ^14^, ^15^

Biologic augmentation may reduce retear risk in tissues with poor vascularity.^16^, ^17^ In rotator‐cuff repairs, the bioinductive collagen patch improves tendon thickness, healing quality, and retear rates.^18^, ^19^ Early hip‐specific results show similar promise, with increased tendon thickness and no patch‐related complications,^20^ supporting its use in high‐grade partial hip abductor tears.

Both open and endoscopic techniques have shown meaningful postoperative improvement following gluteus medius repair, but each offers distinct advantages. Endoscopic repair provides less soft‐tissue disruption and has shown favorable outcomes in partial‐thickness tears.^2^, ^4^ Open repair, however, allows direct visualization of the tendon footprint, more reliable identification of undersurface tear morphology, and precise anchor placement.^5^ Although both approaches achieve comparable functional improvement, delayed recovery and persistent weakness remain concerns in chronic degenerative tears.^6^

The present technique offers several important advantages. By preserving intact bursal‐sided tendon fibers and restoring a broad, anatomic footprint with a trans‐tendon, knotless pulley‐bridge construct, this approach maintains the native length‐tension relationship and enhances compression across the repair site. The addition of a bioinductive collagen scaffold provides further benefits by improving the biologic environment of degenerative gluteal tissue.^20^ Despite these advantages, several limitations should be recognized. Open exposure is more invasive than endoscopic techniques and may carry a small risk of wound‐related complications. Technical challenges also exist, including the potential for suture entanglement and the need for precise anchor placement at the anatomic gluteus medius and minimus footprint. Bone quality may further influence fixation security, with an increased risk of anchor pullout in osteoporotic bone (Table 1). Additional pearls and pitfalls are reported in Table 2. Careful attention to these factors is critical to achieving consistent biologic augmentation and secure mechanical fixation.

**TABLE 1:** Advantages and Disadvantages of the Present Technique

**Advantages**	**Disadvantages**
• Preservation of remaining tendon • Broad footprint compression • Bioinductive collagen scaffold enhances the biologic environment and supports tendon regeneration • Knotless anchors reduce knot irritation and suture bulk at the repair site • Direct visualization (open approach)	• Open technique is more invasive than endoscopic approaches • Higher surgical‐site wound risk compared with endoscopic techniques • Potential for suture tangling, especially with multiple repair and conversion limbs • Osteoporotic bone may predispose to anchor pullout or reduced fixation strength

**TABLE 2:** Pearls and Pitfalls of the Present Technique

**Pearls**	**Pitfalls**
• Anatomically accurate anchor placement • Leave loops open initially to allow the patch to be positioned beneath before tensioning • Independent SutureTape limbs help create strong distal suture‐bridge compression • Up‐size anchors within drilled tunnels to decrease pullout from osteoporotic bone	• Nonanatomic anchor placement • Premature loop tensioning • Increased risk of anchor loosening and repair failure in poor bone quality

In summary, this knotless trans‐tendon pulley‐bridge repair with bioinductive patch augmentation provides a structurally and biologically optimized approach for high‐grade partial gluteus medius and minimus tears. By preserving intact tendon fibers, restoring broad anatomic footprint compression, and enhancing the biologic healing environment, this technique offers a reproducible construct that may improve tendon healing and functional outcomes when meticulous attention is given to anatomic anchor placement and controlled tensioning.

## DISCLOSURES

The author (J.C.) declares the following financial interests/personal relationships which may be considered as potential competing interests: J.C. reports a relationship with American Orthopaedic Society for Sports Medicine that includes: board membership; reports a relationship with Arthrex that includes: consulting or advisory; reports a relationship with Arthroscopy Association of North America that includes: board membership; reports a relationship with Breg that includes: travel reimbursement; reports a relationship with CONMED Linvatec that includes: consulting or advisory; reports a relationship with DePuy Synthes Sales that includes: travel reimbursement; reports a relationship with International Society of Arthroscopy Knee Surgery and Orthopaedic Sports Medicine that includes: board membership; reports a relationship with Joint Restoration Foundation that includes: travel reimbursement; reports a relationship with Medical Device Business Services that includes: travel reimbursement; reports a relationship with Medwest Associates that includes: speaking and lecture fees; reports a relationship with Ossur that includes: consulting or advisory; reports a relationship with Pacira Pharmaceuticals that includes: travel reimbursement; reports a relationship with RTI Surgical that includes: consulting or advisory; reports a relationship with SI‐BONE that includes: travel reimbursement; reports a relationship with Smith & Nephew that includes: consulting or advisory and speaking and lecture fees; reports a relationship with Vericel that includes: consulting or advisory and travel reimbursement. The other authors (M.A.O., N.T., F.C., T.F.V., J.S‐M., T.N.) declare that they have no known competing financial interests or personal relationships that could have appeared to influence the work reported in this paper.
